# Social exclusion affects working memory performance in young adolescent girls

**DOI:** 10.1016/j.dcn.2019.100718

**Published:** 2019-10-16

**Authors:** Delia Fuhrmann, Caroline S. Casey, Maarten Speekenbrink, Sarah-Jayne Blakemore

**Affiliations:** a Institute of Cognitive Neuroscience, University College London, WC1N 3AR, London, UK; b MRC Cognition and Brain Sciences Unit, University of Cambridge, CB2 7EF, Cambridge, UK; c Institute of Neurology, University College London, WC1N 3BG, London, UK; d Department of Experimental Psychology, University College London, WC1H 0AP, London, UK

**Keywords:** Cyberball, Sensitive period, Adolescence, n-back, Visuo-spatial working memory, Mood

## Abstract

Adolescence has been proposed to be a sensitive period of social development, during which the social environment has a heightened effect on brain and behaviour. As such, negative social experiences, such as social exclusion, may have particularly detrimental effects on psychological well-being. However, little is known about how social exclusion affects cognitive performance during this time of life. Here, we compared the effects of exclusion between adolescence and adulthood. We recruited 98 females in three age groups: young adolescents (*N* = 36, aged 10.1–14.0), mid-adolescents (*N* = 35, aged 14.3–17.9) and adults (*N* = 27, aged 18.3–38.1). All age groups showed reductions in mood after exclusion, compared to inclusion, in a virtual ball-tossing game. Young adolescents also showed reduced verbal working memory accuracy following exclusion. There was no effect of exclusion on visuo-spatial working memory in any age group. These results suggest young adolescent girls’ verbal working memory accuracy was affected by a short, virtual social exclusion experience. This highlights the importance of the social environment in adolescence and underlines the need to consider age differences in response to exclusion in the design and timing of social exclusion interventions in schools.

The human brain undergoes protracted development during adolescence, the period of life between the onset of puberty and the point at which we attain an independent role in society (Damon et al., 2004). Adolescence is traditionally thought of as a time of social reorientation during which peers become increasingly important (Crone and Dahl, 2012; Steinberg and Morris, 2001). It has been suggested that adolescence may even be a sensitive period, during which the brain shows heightened plasticity and is particularly susceptible to socio-cultural information (Blakemore and Mills, 2014).

Rodent studies have provided strong evidence that negative social experiences, such as social deprivation, may be especially detrimental during adolescence (Burke et al., 2017; Buwalda et al., 2011; Einon and Morgan, 1977; Fuhrmann et al., 2015). Social isolation in rats has been shown to reduce some aspects of exploratory behaviour, but only if the isolation occurred between postnatal day 25 and 45 (late juvenile to early adolescent stage) – more so than before or after (Burke et al., 2017; Einon and Morgan, 1977). Adolescence, therefore, is thought to constitute a vulnerable period for social deprivation in rats.

Social exclusion can be simulated experimentally in humans using the Cyberball paradigm (Williams et al., 2000). Cyberball is an online ball-tossing game during which the participant is ostensibly either included or excluded by two peers. In adults, the exclusion condition is associated with lower mood, increased anxiety and feeling threatened in four fundamental psychological needs: self-esteem, belonging, control and a sense of meaningful existence (Williams, 2007; Williams et al., 2000). Some studies have suggested that such effects may be amplified in younger age groups. For example, young adolescent girls (aged 11–13) showed a reduction in mood and increase in anxiety after exclusion compared to baseline, while mid-adolescents (aged 14–15) showed reduced mood only, and adults (aged 22–47) showed no changes in either mood or anxiety compared to baseline (Sebastian et al., 2010). Another study showed that Cyberball exclusion threatened psychological needs in adolescents (aged 13–17) and emerging adults (aged 18–22) more than it did in adults (aged 22–27; Pharo et al., 2011). A meta-analysis, however, showed that exclusion generally has large (d > |1.4|) effects on intrapersonal outcome measures such as self-esteem, regardless of age (Hartgerink et al., 2015).

Social exclusion may affect not only mood, anxiety and need-threat but also cognitive performance (Baumeister et al., 2002). The mechanisms underlying this effect are, at present, unclear. Candidate mechanisms include ruminative thought following exclusion disrupting cognitive performance, or emotion regulation interfering with cognitive control (Curci et al., 2013; Hofmann et al., 2012). Studies using the Cyberball paradigm in adults have largely found deleterious effects of exclusion particularly on executive functions such as inhibitory control and working memory. For instance, Cyberball exclusion was associated with reduced performance in the Flanker task (Themanson et al., 2014) and the anti-saccade task (Jamieson et al., 2010) in adults. Cyberball has also been shown to disrupt cognitive performance in children. Hawes and colleagues showed that social exclusion disrupted cognitive performance in girls, but not boys, aged 8–12 (Hawes et al., 2012). To date, however, there is little experimental evidence on the effects of social exclusion on cognitive performance in adolescence.

The aim of the current study was to address this developmental gap and investigate the effects of experimentally-induced social exclusion on cognitive performance during adolescence, so as to gain a better understanding of the cognitive ramifications of social exclusion in schools. Social exclusion and bullying are relatively common experiences in childhood and adolescence. Around 34% of school-age children are bullied (Forero et al., 1999). Bullying is longitudinally associated with lasting effects on mental health (Arseneault et al., 2010). Correlational studies have linked bullying to reduced educational attainment (Rigby, 2000; Sharp, 1995; Sigurdson et al., 2015). Social exclusion is a common form of bullying, particularly in girls (Wang et al., 2010). Longitudinal evidence suggests strong, mostly bidirectional effects between peer-relations and executive functions, not only in laboratory tasks but also naturalistic observations (de Wilde et al., 2016; Holmes et al., 2016)

To investigate age-related differences in the effects of social exclusion on cognitive processing, we compared the impact of exclusion on working memory and mood in 98 female adolescents and adults. We chose to recruit females only because adolescent girls have been found to spend more time with peers than boys (Larson and Richards, 1991) and have more one-to-one peer relationships, compared to boys, who generally have more interconnected group relationships (Benenson and Christakos, 2003), potentially making peer-rejection more relevant to girls. In childhood, girls have also been shown to be more sensitive to social exclusion than boys (Hawes et al., 2012 but see Sandstrom et al., 2017). In adulthood, women tend to use and experience indirect forms of social aggression, such as exclusion, more frequently than men (Benenson et al., 2011; Benenson et al., 2013).

Participants were divided into three age groups: young adolescents (*N* = 36, aged 10.1–14.0), mid-adolescents (*N* = 35, aged 14.3–17.9) and adults (*N* = 27, aged 18.3–38.1). Adolescent participants were divided into two age groups because previous research suggested that socio-cognitive functions and their neural substrates change between early and mid-adolescence (Blakemore and Mills, 2014; Knoll et al., 2015; Scherf et al., 2012). For instance, peer influence was found to peak between ages 11–14 (Berndt, 1979) and young adolescents (aged 11–13) may show more anxiety after Cyberball exclusion than mid-adolescents (aged 14–15) (Sebastian et al., 2010). These differences between young and mid adolescents may be driven by changes in social networks during early adolescence, which in turn may partly be due to the transition from primary to secondary school (Burnett Heyes et al., 2015; Cantin and Boivin, 2004). Participants over the age of 18 were qualitatively different from our adolescent participants in that they were not recruited and tested in schools, and were therefore allocated to their own age group.

Participants experienced the inclusion and exclusion condition in the Cyberball game. After each Cyberball condition, participants completed a mood questionnaire, as well as working memory tasks. We chose working memory tasks as indicators of cognitive performance because working memory is educationally relevant. It is closely related to other important cognitive functions such as fluid intelligence (Kane et al., 2004). In addition, working memory is predictive of academic performance (Alloway and Alloway, 2010; Alloway et al., 2009; Gathercole et al., 2003). Working memory performance at age five, for instance, is one of the strongest predictors of literacy and numeracy up to six years later (Alloway and Alloway, 2010). Using working memory tasks also allowed us to compare our results to previous Cyberball studies using working memory and other executive function tasks in children and adults (Hawes et al., 2012; Jamieson et al., 2010; Themanson et al., 2014). We assessed both working memory updating performance (n-back) and visuo-spatial span (dot-matrix) working memory as updating and complex span performance are proposed to rely on separate, partly dissociable systems (Schmiedek et al., 2009). Assessing both components can therefore give a more complete picture of working memory performance. We also explored whether the effects of social exclusion would be more evident on complex versions of each working memory task, as suggested by Baumeister et al. (2002), or on simple versions of these tasks, similarly to previous findings by Hawes et al. (2012).

We hypothesized that social exclusion would reduce n-back and dot-matrix task performance across age groups, and that this effect would decrease from adolescence to adulthood. In line with previous studies (Einon and Morgan, 1977; Sebastian et al., 2010; Pharo et al., 2011), we also expected that social exclusion would be associated with lower mood in all age groups, and that effects would be stronger in adolescents than in adults.

## 1 Methods

### 1.1 Participants

One-hundred and thirteen female participants aged 10–38 years were recruited for the purpose of this study. Adolescent participants were recruited from seven secondary schools (two state, four private and one grammar) in London and Oxfordshire and tested individually in schools. Adult participants were recruited from University College London (UCL) participant pools and tested in the lab. UCL participant pools consist of members of the general public, not just students. A researcher tested each participant individually in a quiet room. Six participants were excluded from all analyses because they reported psychiatric or developmental disorders, one because they scored below 70 IQ points, two because of technical difficulties during testing and six because they didn’t believe the Cyberball manipulation (see Materials section). The remaining 98 participants were allocated to one of three age groups: young adolescents, mid-adolescents and adults (Table 1). To ensure that our main finding was not an artefact of allocating participants to age groups, we replicated the effect using age as a continuous variable (Supplementary Material).

**Table 1:** Participant Characteristics.

Age				
*Age group*	*Min*	*Max*	*M*	*SE*
Young adolescents	10.13	14.03	12.87	0.14
Mid-adolescents	14.27	17.87	15.93	0.19
Adults	18.34	38.14	24.93	0.91

**IQ**				
*Age group*	*Min*	*Max*	*M*	*SE*
Young adolescents	71.54	119.51	101.38	1.89
Mid-adolescents	74.54	113.51	98.43	1.74
Adults	77.54	133.00	108.02	2.63

**SES**				
*Age group*	*Min*	*Max*	*Median*	*IQR*
Young adolescents	1	6	5	0.75
Mid-adolescents	1	6	5	2.00
Adults	1	6	5	1.75

*Note.* SES = socio-economic status; IQR = interquartile range; IQ was measured by matrix reasoning tests (Wechsler, 1999); SES was measured by parental education for all age groups. Parental education is a robust indicator of SES (Dubow et al., 2009). SES scores: 1 = 1 + O levels/ CSEs/GCSEs; 2 = 5 + O levels/CSEs/CSEs; 3 = 1 + A levels/AS levels; 4 = 3 + A levels/AS levels; 5 = First Degree (e.g. BA, BSc); 6 = Higher Degree (e.g. MA, PhD).

We examined socio-economic status (SES) and IQ differences between age groups as potential confounds. IQ was measured by matrix reasoning tests (WASI; Wechsler, 1999). We analysed this data in two ways: IQ scores (reported in the main manuscript) and matrix reasoning raw scores (reported in the Supplementary Material). The results for our main analyses were robust across these two scoring methods (Supplementary Analyses).

There was no significant difference between age groups in terms of SES (*χ*^2^(2) = 3.50, *p* =  0.174), but IQ showed differences between age groups overall (*F*(2, 90) = 5.16, *p* =  0.008). Post-hoc tests showed that IQ did not differ significantly between young adolescents and mid-adolescents (*t*(90) = 1.02, *p_Bonf._* = 0.930) or young adolescents and adults (*t*(90) = -2.17, *p_Bonf._* = 0.098). It did, however, differ between mid-adolescents and adults (*t*(90) = -3.17, *p_Bonf._* = 0.006). Using WASI raw scores, differences were significant for the contrast between young-adolescents and adults only (*t*(90) = -2.99, *p_Bonf._* = 0.011). Because of these differences, IQ was controlled for in all analyses (see Design and Analysis section).

The study was carried out in accordance with the UCL Research Ethics Guidelines and was approved by the UCL Research Ethics Committee (project number: 3453/001, project title: Development of cognitive processing during adolescence). Informed consent was obtained from adult participants and from parents of participants under 18, who provided written assent.

### 1.2 Materials

#### 1.2.1 Cyberball

Social inclusion and exclusion were simulated using the freeware Cyberball 4.0 program (Williams et al., 2000). This program features two virtual players who played an online ball-tossing game lasting ∼2 min with the participant. Whilst participants were told the other players were real, and like themselves, participating in the study and connected to them via the internet, the Cyberball players were in fact programmed to either include or exclude the participant from the game. Inclusion generated one third of the ball tosses to the participant. Exclusion generated only two tosses to the participant at the beginning of the game, after which the other players no longer threw the participant the ball.

To check whether participants believed the other players to be authentic, we asked three questions during the debrief after the experiment (Will et al., 2016):

1 What did you think of the Cyberball game?
2 How did you like being connected to other people though the internet?
3 What did you think the study was about?

We recorded whether or not participants voiced suspicion about authenticity during this probe. Six participants (three mid-adolescents and three adults) voiced that they thought the other players were not real, and were therefore excluded from all analyses.

#### 1.2.2 Working memory measures

All participants completed two different measures of working memory: an n-back working memory task and a dot-matrix visuo-spatial working memory task. The order of these tasks was counterbalanced between participants. The first 20 participants also completed a digit span task. This task was then cut from the procedure because of time constraints in schools, and data from this task were not analysed. All tasks were programmed in Cogent (Cogent 2000 team, 2015) and MATLAB (The Mathworks, Inc., 2013) and accuracy (correct/incorrect) and response times for each task were recorded.

#### 1.2.3 N-back task

In the n-back working memory task (Gevins and Cutillo, 1993), numbers were flashed one-by-one on a screen for 500 ms with a variable delay in between (1000–3000 ms, mean delay: 2000 ms). The task required participants to indicate whether the current number on the screen was i) a zero (0-back task) or ii) the same as the number that appeared "two back" in the sequence (2-back task). Distractors were shown simultaneously with the number. Distractors consisted of photos of a house, a happy face or a fearful face. They appeared on both sides of the number and were added to vary the affective context of the task. Participants were instructed to ignore them. Participants completed six blocks of 12 trials each. Half of these blocks were 0-back tasks, half were 2-back tasks. The order of blocks and response buttons was counterbalanced between participants.

#### 1.2.4 Dot-matrix task

The dot-matrix task is a visuo-spatial working memory task (Alloway et al., 2009). Participants were shown a four-by-four white grid on a black background. Dots were flashed one-by-one for 300 ms and with a 600 ms delay in between. Dots were displayed in any of the 16 squares of the grid. After all dots in a particular sequence were shown, the grid turned orange for 1500 ms, then turned white again. Participants were instructed to click on the fields of the grid where the dots had appeared; and in the order they had appeared. Sequence length increased from three to eight dots. Three sequences of each length were shown.

#### 1.2.5 Questionnaire measures

Participants were administered a standard mood and need-threat questionnaire after each Cyberball condition (Williams et al., 2000). We analysed the mood questionnaire here in which participants rated how good/bad, happy/sad, friendly/unfriendly and relaxed/tense they were currently feeling, on a scale of 1 (not at all) - 5 (very much). Negative items were re-coded. Based on previous studies (Williams et al., 2000; Sebastian et al., 2010), we calculated an average mood rating for each participant and each Cyberball condition.

### 1.3 Procedure

Participants practised the two working memory tasks at the beginning of the experiment. They were then introduced to Cyberball. All participants played Cyberball twice and experienced both inclusion and exclusion. The order of the Cyberball conditions was counterbalanced between participants. Participants completed the mood questionnaire and two working memory tests after each Cyberball condition. Participants were then fully debriefed. The experiment took ∼60 min in total.

### 1.4 Design and analysis

We used a 2 × 3 mixed design with Cyberball condition (inclusion/exclusion) as the within subjects measure and age group (young adolescent/mid-adolescent/adult) as the between subjects measure.

Data were analysed using Generalized Linear Mixed-Models (GLMMs) in R (R core team, 2015) and lme4 (Bates et al., 2013). GLMMs are a flexible, regression-based approach that allowed us to model binary accuracy data (correct/incorrect) as well as continuous response times and mood ratings.

For each of the working memory tasks, we specified one model for accuracy and one for response times. Accuracy was analysed as a binary dependent variable (correct/incorrect) and modelled using the binomial distribution. Response times and mood ratings were each averaged over Cyberball condition for each participant and analysed as continuous dependent variables. In all of these models, Cyberball condition, age group and the interaction between the two were specified as orthogonal, Helmert-coded fixed effects. IQ was included as a z-scored covariate and participant number and school as a nested random intercept. To assess our hypotheses, we planned contrasts of exclusion and inclusion within and between age groups *a priori*. We inspected contrasts within age groups using lsmeans (Lenth, 2016). To inspect contrasts between age groups we ran additional models for all dependent variables. These models were identical to those described above but included age group as a dummy-coded variables. Changing the reference group for age then allowed us to compare all age groups to one another (Fuhrmann et al., 2016). We Bonferroni-corrected for three comparisons in each set of contrasts.

In an exploratory analysis, we specified four additional models predicting accuracy and response times for the n-back and dot-matrix task each. For the n-back task, models were specified as described above but additionally included task difficulty (0-back/2-back) and distractor type (happy face/fearful face/house) as Helmert-coded fixed effects. For the dot-matrix task, two models were specified as described above but task difficulty (low: 3–5 dots / high: 6–8 dots) was included as an additional factor. All models also included all possible interactions between the fixed effects.

## 2 Results

We analysed age-dependent effects of Cyberball exclusion on working memory performance and mood using GLMMs. We took a four-stepped approach for our main analyses. First, we inspected whether there was a main effect of Cyberball condition to assess whether there was an overall difference in performance between inclusion and exclusion. Second, we inspected the interaction between Cyberball condition and age to understand whether the effect of Cyberball was moderated by participants’ age. As a third and fourth step, we probed this interaction further using two sets of planned contrasts. One set of planned contrasts assessed whether there were differences in between exclusion and inclusion *within* each age group. The second set of planned contrasts assessed whether potential differences in between exclusion and inclusion differed *between* age groups.

### 2.1 Working memory performance

We assessed accuracy and response times in the n-back working memory task and dot-matrix visuo-spatial working memory task.

#### 2.1.1 N-back task

##### 2.1.1.1 Accuracy

There was no main effect of Cyberball condition for n-back accuracy (*χ^2^*(1) = 0.17, *p* = 0.677), indicating that there was no overall difference in performance between inclusion and exclusion (Table 2).

**Table 2:** Overall Performance in the N-Back and Dot-Matrix Task.

	N-back				Dot-matrix			
Cyberball condition	probability of correct responses		RT (ms)		probability of correct responses		RT (ms)	
	*Mean*	*SE*	*Mean*	*SE*	*Mean*	*SE*	*Mean*	*SE*
Inclusion	0.96	0.004	777.34	18.39	0.70	0.02	3434.95	100.44
Exclusion	0.96	0.005	781.40	18.38	0.69	0.02	3512.03	100.53

*Note.* RT = response times. Model-predicted values are shown.

**Fig. 1:**
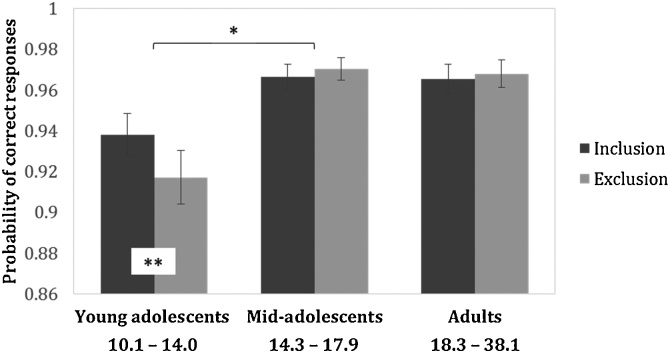
N-back accuracy after inclusion and exclusion. Mean probability of correct responses with standard error bars are shown for three age groups: young adolescents, mid-adolescents and adults. All values shown are model-predicted. Asterisks at the bottom of the bars in white boxes indicate significant differences between Cyberball conditions within a particular age group. Asterisks above the bars indicate that such effects differed between age groups. * p_Bonf._ < 0.05; ** *p*_Bonf._ < 0.01.

However, there was a significant interaction between Cyberball condition and age group (*χ^2^*(2) = 7.87, *p* = 0.020). Planned within-age-group contrasts showed that young adolescents were the only age group to show reduced n-back accuracy after exclusion compared to inclusion. Mid-adolescents and adults showed no significant difference between exclusion and inclusion (Fig. 1; Supplementary Table 1). Planned between-age-group contracts showed that the reduction in n-back accuracy in young adolescents was significantly greater than differences between exclusion and inclusion in mid-adolescents. The difference between young adolescents and adults did not survive correction for multiple comparison (Fig. 1; Supplementary Table 2). Overall, this shows that the effects of Cyberball exclusion on n-back accuracy were moderated by age with younger adolescents being most detrimentally affected. We note, however, that the overall interaction effect was not significant when modelling age as a continuous variable (*p* =  0.058; Supplementary Analyses).

There was a significant 3-way interaction between Cyberball condition, age group and task difficulty (*χ^2^*(2) = 7.96, *p* = 0.019). This indicated that the age differences in response to exclusion were moderated by task difficulty (0-back or 2-back). Post-hoc tests showed that young adolescents showed a greater reduction in performance in response to exclusion on the 0-back than the 2-back task (*z* = -3.07, *p*_Bonf._ = 0.007). There was no difference between the 0- and 2-back task for any of the other age groups (mid-adolescents: *z* = 1.12, *p*_Bonf._ = 0.785; adults: *z* = 0.07, *p*_Bonf._ = 1). These age-group differences were unlikely to be due to ceiling effects as all age groups performed significantly below 100% (Supplementary Table 3). There was no significant interaction between Cyberball condition, age group and distractor type (happy face, fearful face, or house; (*χ^2^*(4) = 3.01, *p* = 0.557).

##### 2.1.1.2 Response times

There was no main effect of Cyberball condition for n-back response times (*χ^2^*(1) = 0.20, *p* = 0.656), indicating that overall, there was no difference in performance between exclusion and inclusion (Table 2). There was also no significant interaction between Cyberball condition and age group (*χ^2^*(2) = 3.27, *p* = 0.195) and planned comparisons showed that there were no significant differences between Cyberball exclusion and inclusion for any of the three age groups (Supplementary Table 1; Supplementary Table 2). There was also no significant interaction between Cyberball condition, age group and task difficulty (χ^2^(2) = 3.46, *p* = 0.178) or distractor type (χ^2^(4) = 2.00, *p* = 0.735).

#### 2.1.2 Dot-matrix task

##### 2.1.2.1 Accuracy

There was no main effect of Cyberball condition for dot-matrix accuracy (*χ^2^*(1) = 0.42, *p* = 0.516), indicating that overall performance was matched between exclusion and inclusion (Table 2). The interaction between Cyberball condition and age group was not significant (*χ^2^*(2) = 0.44, *p* = 0.802). Accuracy did not differ significantly between inclusion and exclusion for any age group (Supplementary Table 1; Supplementary Table 2). There was also no significant 3-way interaction between Cyberball condition, age group and task difficulty (low: 3–5 dots / high: 6–8 dots) for dot-matrix accuracy (*χ^2^*(2) = 2.18, *p* = 0.337).

##### 2.1.2.2 Response times

There was no main effect of Cyberball condition for dot-matrix response times (*χ^2^*(1) = 1.61, *p* = 0.205), indicating that overall performance did not differ between exclusion and inclusion (Table 2). The interaction between Cyberball condition and age group was not significant (*χ^2^*(2) = 3.70, *p* = 0.158). Planned comparisons showed an increase in dot-matrix response times after exclusion compared to inclusion in young adolescents, but this effect did not survive Bonferroni correction (*t*(88.09) = 2.26, *p*_Bonf._ = 0.079; Supplementary Table 1). There was no significant 3-way interaction between Cyberball condition, age group and task difficulty (*χ^2^*(2) = 1.77, *p* = 0.413).

### 2.2 Mood ratings

**Fig. 2:**
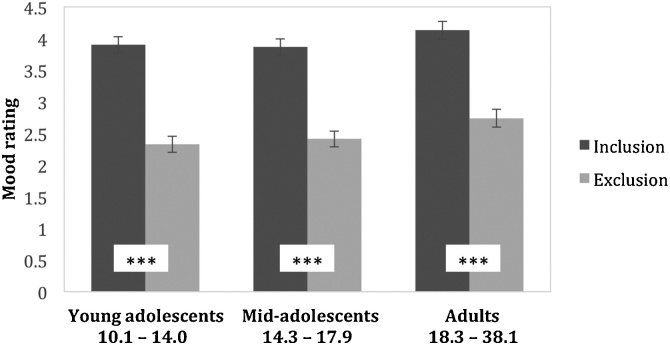
Mood ratings after inclusion and exclusion. Mean ratings with standard error bars are shown for three age groups: young adolescents, mid-adolescents and adults. All values shown are model-predicted. Asterisks in white boxes at the bottom of the bars indicate significant differences between Cyberball conditions within a particular age group. None of the comparisons between age groups were significant. *** *p*_Bonf._ < 0.001.

We analysed participants’ mood ratings after inclusion and exclusion in the Cyberball game using Linear Mixed Models. There was a significant main effect of Cyberball condition on mood (*χ^2^*(1) = 212.04, *p* < 0.001). Mood was lower after exclusion (*M* = 2.49, *SE* = 0.08) compared to inclusion (*M* = 3.97, *SE* = 0.08) overall. This effect did not differ between age groups (*χ^2^*(2) = 0.53, *p* = 0.765), however (Fig. 2; Supplementary Table 1; Supplementary Table 2). To further probe the relationship between Cyberball exclusion, mood and working memory, we carried out an exploratory analysis to test whether the effects of Cyberball on working memory were moderated by mood. We found no evidence for a moderation in any of our outcome measures (Supplementary Table 4). This finding supports the notion that the effects of Cyberball on working memory were independent from the emotional effects of exclusion.

## 3 Discussion

In the current study, we investigated the impact of social exclusion on cognitive performance and mood in three age groups: young adolescents (aged 10.1–14.0), mid-adolescents (aged 14.3–17.9) and adults (aged 18.3–38.1). While all age groups showed a similar and significant reduction in mood after social exclusion, the effect of exclusion on cognitive performance was age-dependent. Only young adolescents showed a reduction in verbal working memory accuracy after social exclusion; this was not the case for mid-adolescents or adults. There was no effect of exclusion on visuo-spatial working memory in any age group. These findings suggest that some aspects of young adolescents’ cognitive performance may be particularly sensitive to social exclusion.

Previous research showed negative effects of Cyberball exclusion on executive functions in adults (Jamieson et al., 2010; Lustenberger and Jagacinski, 2010; Themanson et al., 2014) and working memory in children (Hawes et al., 2012). Based on this literature, we hypothesized that all age groups would show reductions in cognitive performance after exclusion, but expected the effect to be more pronounced in adolescents. While n-back working memory performance was reduced after exclusion in younger adolescents, we found no effect of social exclusion on working memory performance in mid-adolescents or adults. It is possible that the effects of social exclusion depend on the specific executive function tasks used. Executive functions are proposed to decompose into rule-driven, explicit and internalized, automatic processes (Crone and Steinbeis, 2017; Olsson and Ochsner, 2008). Effects may be stronger when tasks require internalized, automatic executive function processes such as inhibition in the anti-saccade task (Jamieson et al., 2010) or the Flanker task (Themanson et al., 2014). Such automatic processes might be relatively inflexible and easily disrupted by stressful situations, particularly in younger age groups. In contrast, rule-driven and explicit tasks may allow participants, and particularly older adolescents and adults, to allocate cognitive resources more dynamically under changing situational demands (but see Baumeister et al., 2002). We saw some evidence for such a dissociation, in that effects were mainly evident on the quick-paced n-back task, and not on the self-paced dot matrix task. This explanation remains speculative, however, and needs to be tested in future research.

Contrary to our hypothesis, we found no significant age group differences in mood: all age groups showed similar significant reductions after social exclusion. While this finding is dissimilar to some previous studies on mood, anxiety and need-threat (Pharo et al., 2011; Sebastian et al., 2010), it is line with a recent meta-analysis of 120 Cyberball studies. This meta-analysis showed that the emotional effects of exclusion are mostly independent of age (Hartgerink et al., 2015). The absence of age-dependent effects on mood suggests it is unlikely that the effects of Cyberball of working memory performance reported here were due to differences between age groups in the emotional response to Cyberball. All three age groups showed similar mood reductions after exclusion and yet n-back working memory performance was affected in young adolescents only. This indicates that the age-dependent effects of Cyberball were relatively specific to working memory performance. This finding is in line with previous studies in adults showing that the effects of social exclusion on cognitive performance are not mediated by mood (Baumeister et al., 2002; Buckley et al., 2004). Instead, self-regulatory processes such as suppression of ruminative thought or active down-regulation of unwanted affect may be candidate mechanisms for the reduction in working memory performance (Curci, et al., 2013; Hofmann et al., 2012). These self-regulatory processes are thought to interact and compete with executive functions (Hofmann et al., 2012).

Our analysis showed that the effect of exclusion in young adolescents was stronger for easy trials (0-back) than for difficult trials (2-back) in the n-back task. First, this dissociation suggests that the age-dependent effects of social exclusion reported here were not just due to the protracted development of working memory in adolescence. Second, this result challenges ego-depletion as a mechanistic explanation for the effects of social exclusion on cognitive performance (Baumeister et al., 2002). Instead, it replicates the results of a previous Cyberball study in which girls aged 8–12 also showed reduced cognitive functioning on easy but not hard working memory tasks (Hawes et al., 2012). A possible mechanism for this pattern of results is that the easier 0-back trials may have allowed for more rumination than the demanding 2-back trials. Rumination, in turn, is known to increase the emotional impact of Cyberball exclusion (Wesselmann et al., 2013) and also to disrupt cognitive performance (Curci et al., 2013; Hofmann et al., 2012). Conversely, demanding cognitive tasks have been shown to decrease the incidence of traumatic flashbacks (Holmes et al., 2009). There was no difference between easy and hard trials in the visuo-spatial working memory test. This task was self-paced, which may have allowed for similar rumination on all types of trials.

There are several limitations of the study. IQ differed between age groups such that adults had higher IQ than the other two age groups. We think it unlikely, however, that IQ could explain stronger performance reductions after social exclusion in younger adolescents because IQ was controlled for in all analyses. Next, we cannot rule out the possibility that there may have been age differences in the perception of the authenticity of Cyberball. The similarity of the mood response in all age groups suggests that this is unlikely, however. Moreover, we carried out a three question probe during debrief as described by Will et al. (2016) and excluded those participants who said they did not believe the manipulation. It is possible however, that more quantitative methods could have picked up on more subtle differences between age groups. We also note that the age-dependent effects of social exclusion on cognitive performance were mainly evident in the n-back working memory task, in which young adolescents showed a reduction in accuracy that was significant at the interaction level. This interaction effect became non-significant (*p* =  .058) when age was analysed as a continuous variable, indicating that it should be interpreted with caution. Another limitation is that our study included only female participants and we do not, at present, know whether our results would generalize to males. Adolescent girls may spend more time with peers than boys (Larson and Richards, 1991) and may be particularly sensitive to social exclusion (see Hawes et al., 2012 for evidence in children). It is thus important for future research to explore whether adolescent boys react differently to social exclusion than adolescent girls. Another avenue for future research is exploring the effects of puberty. This developmental metric may explain additional variance in development over and above age (Goddings et al., 2014). Puberty may also prove particularly informative from a theoretical perspective because puberty may trigger the onset of sensitive periods (Fawcett and Frankenhuis, 2015). Finally, social exclusion simulations such as Cyberball are likely to evoke only temporary effects. Zadro and colleagues showed that the emotional effects of Cyberball exclusion mostly dissipated over the course of 45 min, although they may persist longer in socially-anxious participants (Zadro et al., 2006). The experience of exclusion in real-life settings is likely to be repeated and more personal and therefore also likely more profound and potentially longer lasting. This is reflected in the body of evidence from observational studies highlighting the link between bullying, mental health and cognitive performance across ages (Arseneault et al., 2010; Rigby, 2000; Sharp, 1995; Sigurdson et al., 2015).

Overall, our results indicate that young adolescent girls’ verbal working memory may be susceptible to the effects of a short virtual social exclusion experience. There was no evidence for effects on visuo-spatial working memory. This extends previous research showing that children aged 8–12 were affected by social exclusion (Hawes et al., 2012) by showing a similar result for young adolescents. This sensitivity to social exclusion in late childhood and early adolescence is in line with rodent studies, which have shown a sensitive period for social isolation during the late juvenile and early adolescent stage (Einon and Morgan, 1977). Future research could investigate effects of social exclusion in humans across a broader age range, particularly earlier in childhood, to explore whether there is a peak of sensitivity to social exclusion in late childhood and early adolescence, as there is in rodents (Buwalda et al., 2011; Einon and Morgan, 1977), or whether sensitivity to exclusion simply decreases over development (e.g. see Ladd, 2006).

## Author Contributions

DF and SJB designed the study. DF and CSC collected the data. DF, CSC and MS analysed the data and all authors contributed to the writing of the manuscript.

## Declaration of Competing Interests

The authors declare that they have no known competing financial interests or personal relationships that could have appeared to influence the work reported in this paper.
