# Supplementary material for: Social exclusion affects working memory performance in young adolescent girls
Source: Dev Cogn Neurosci. 2019 Oct 16;40:100718. doi: 10.1016/j.dcn.2019.100718 (PMC6905155; doi:10.1016/j.dcn.2019.100718)
Supplement: Supplementary file 1 [file mmc1.pdf]

Supplementary Material

Supplementary Analyses

Age as a continuous variable

To test whether our findings were an artefact of our *a priori* decision to analyse age as a categorical variable, we re-ran our models with age as a continuous predictor. Based on the non-linear pattern we observed in our main analysis (performance reductions after exclusion mainly in early adolescence, no reductions in older age groups) we log-transformed age and then centred it to participants average age.

We found no significant interaction effect between age and Cyberball condition for n-back accuracy ($\chi^2(1)= 3.59$, $p = .058$). This, therefore indicates, that the reduction in performance after exclusion observed in our main analysis may not be robust across analyses methods.

For all other models, the interaction between age and Cyberball condition remained non-significant (n-back response times: $\chi^2(1)= 0.45$, $p = .501$; dot-matrix accuracy: $\chi^2(1)= 0.002$, $p = .964$; dot-matrix response times: $\chi^2(1)= 1.15$, $p = .284$; mood: $\chi^2(1)= 0.51$, $p = .475$).

IQ modelled as a WASI matrix reasoning raw score

We tested if results depended on whether IQ was controlled as a transformed t-score (as reported in the main analysis) or as a WASI matrix reasoning raw score (reported here). The results were robust across these two analyses methods.

We found a significant interaction effect between age and Cyberball condition for n-back accuracy ($\chi^2(2)= 7.87$, $p = .020$). The reduction in n-back accuracy after exclusion in young adolescents reported in our main analysis was significant ($z = -2.96$, $p_{\text{Bonf.}} = .009$) and significantly greater than the effect in mid-adolescents ($z = -2.47$, $p_{\text{Bonf.}} = .040$). For all other models, the interaction between age and Cyberball condition remained non-significant (n-back response times: $\chi^2(2)= 3.26$, $p = .196$; dot-matrix accuracy: $\chi^2(2)= 0.44$, $p = .802$; dot-matrix response times: $\chi^2(2)= 3.69$, $p = .\ 158$; mood: $\chi^2(2)= 0.53$, $p = .\ 765$).

Median response times

To test if our decision to analyse mean response times on correct trials affected our results, we re-ran our models with median response times on correct trials as the dependent variable. Results were very similar for mean and median response times: none of the main effects and interactions reached significance for the n-back task (Cyberball condition main effect: $\chi^2(1)= 1.50$, $p = .221$; interaction with age: $\chi^2(2)= 2.54$, $p = .281$) or dot-matrix task (Cyberball condition main effect: $\chi^2(1)= 3.02$, $p = .082$; interaction with age: $\chi^2(2)= 3.57$, $p = .168$). We note that there was a significant increase in response times after exclusion in young adolescents in the dot-matrix task ($t(88.09) = 2.56$, $p_{\text{Bonf.}} = .037$).

Supplementary Tables

Supplementary Table 1

Cognitive Performance and Mood Compared between Exclusion and Inclusion within Age Groups

**N-back accuracy**						
Contrast	*Odds ratio*	*SE*	*z*		*p*	$p_{\text{Bonf.}}$
Young adolescents	0.73	0.08	-2.96		.003	.009
Mid-adolescents	1.14	0.17	0.91		.363	1
Adults	1.08	0.17	0.50		.616	1
**N-back response times**						
Contrast	*Estimate*	*SE*	*df*	*t*	*p*	$p_{\text{Bonf.}}$
Young adolescents	-0.17	15.17	86.02	-0.01	.991	1
Mid-adolescents	26.21	15.61	87.41	1.68	.097	.290
Adults	-13.86	16.52	86.02	-0.84	.404	1
**Dot-matrix accuracy**						
Contrast	*Odds ratio*	*SE*	*z*		*p*	$p_{\text{Bonf.}}$
Young adolescents	0.99	0.13	-0.07		.943	1
Mid-adolescents	0.89	0.12	-0.92		.356	1
Adults	0.98	0.14	-0.14		.889	1
**Dot-matrix response times**						
Contrast	*Estimate*	*SE*	*df*	*t*	*p*	$p_{\text{Bonf.}}$
Young adolescents	236.75	104.67	88.09	2.26	.026	.079
Mid-adolescents	43.39	98.66	87.1	0.44	.661	1
Adults	-48.89	112.37	88.26	-0.44	.665	1
**Mood ratings**						
Contrast	*Estimate*	*SE*	*df*	*t*	*p*	$p_{\text{Bonf.}}$
Young adolescents	-1.57	0.17	90	-9.16	<.0001	<.001
Mid-adolescents	-1.45	0.17	90	-8.73	<.0001	<.001
Adults	-1.39	0.19	90	-7.45	<.0001	<.001

Supplementary Table 2

Cognitive Performance and Mood Compared between Exclusion and Inclusion between Age Groups

**N-back accuracy**						
Contrast	*Estimate*	*SE*		*z*	*p*	$p_{\text{Bonf.}}$
Young adolescents vs. mid-adolescents	-0.22	0.09		-2.47	.013	.040
Young adolescents vs. adults	-0.20	0.10		-2.06	.040	.119
Mid-adolescents vs. adults	0.03	0.11		0.25	.806	1
**N-back response times**						
Contrast	*Estimate*	*SE*	*df*	*t*	*p*	$p_{\text{Bonf.}}$
Young adolescents vs. mid-adolescents	-13.19	10.88	86.86	-1.21	.229	.686
Young adolescents vs. adults	6.84	11.21	86.15	0.61	.543	1
Mid-adolescents vs. adults	20.04	11.36	86.76	1.76	.081	.244
**Dot-matrix accuracy**						
Contrast	*Estimate*	*SE*		*z*	*p*	$p_{\text{Bonf.}}$
Young adolescents vs. mid-adolescents	0.06	0.09		0.61	.543	1
Young adolescents vs. adults	0.01	0.09		0.05	.957	1
Mid-adolescents vs. adults	-0.05	0.10		-0.53	.596	1
**Dot-matrix response times**						
Contrast	*Estimate*	*SE*	*df*	*t*	*p*	$p_{\text{Bonf.}}$
Young adolescents vs. mid-adolescents	96.68	71.91	89.92	1.34	.182	.547
Young adolescents vs. adults	142.82	76.77	90.47	1.86	.066	.198
Mid-adolescents vs. adults	46.14	74.75	90.04	0.62	.539	1
**Mood ratings**						
Contrast	*Estimate*	*SE*	*df*	*t*	*p*	$p_{\text{Bonf.}}$
Young adolescents vs. mid-adolescents	-0.06	0.12	90	-0.50	.622	1
Young adolescents vs. adults	-0.09	0.13	90	-0.71	.479	1
Mid-adolescents vs. adults	-0.03	0.12	90	-0.25	.805	1

Supplementary Table 3

Mean Accuracy in the N-Back Task

Age group	*Cyberball condition*	*Mean accuracy*	*SE*	*One-sample t-test (comparing mean to 1)*			
				t	*df*	*p*	$p_{Bonf.}$
**0-Back task**							
Young adolescents	*Exclusion*	0.92	0.03	-2.84	35	.0075	.045
Young adolescents	*Inclusion*	0.95	0.01	-3.94	35	.0004	.002
Mid-adolescents	*Exclusion*	0.98	0.00	-4.35	32	.0001	.001
Mid-adolescents	*Inclusion*	0.97	0.00	-5.68	32	<.0001	<.001
Adults	*Exclusion*	0.97	0.01	-3.44	26	.0020	.012
Adults	*Inclusion*	0.97	0.01	-4.29	26	.0002	.001
**2-Back task**							
Age group	*Cyberball condition*	*Mean accuracy*	*SE*	*One-sample t-test (comparing mean to 1)*			
				t	*df*	*p*	$p_{Bonf.}$
Young adolescents	*Exclusion*	0.86	0.03	-4.40	35	<.0001	<.001
Young adolescents	*Inclusion*	0.87	0.02	-7.12	35	<.0001	<.001
Mid-adolescents	*Exclusion*	0.93	0.01	-5.19	32	<.0001	<.001
Mid-adolescents	*Inclusion*	0.94	0.01	-5.15	32	<.0001	<.001
Adults	*Exclusion*	0.93	0.02	-3.36	26	.0024	.014
Adults	*Inclusion*	0.94	0.02	-3.16	26	.0040	.024

Note. P-values smaller than .05 suggest that accuracy was significantly below 1.

Supplementary Table 4
Moderation Effects of Mood

	χ^2	*df*	*p*
n-back accuracy			
Cyberball Condition : Mood	0.71	1	.399
Cyberball Condition : Age Group : Mood	0.10	2	.953
n-back response times			
Cyberball Condition : Mood	0.35	1	.554
Cyberball Condition : Age Group : Mood	3.38	2	.185
dot-matrix accuracy			
Cyberball Condition : Mood	0.12	1	.733
Cyberball Condition : Age Group : Mood	0.60	2	.740
dot-matrix response times			
Cyberball Condition : Mood	0.36	1	.548
Cyberball Condition : Age Group : Mood	0.24	2	.888
